# *Radix Puerarin* Extract (*Puerarin*) Could Improve Meat Quality of Heat-Stressed Beef Cattle Through Changing Muscle Antioxidant Ability and Fiber Characteristics

**DOI:** 10.3389/fvets.2020.615086

**Published:** 2021-01-15

**Authors:** Yanjiao Li, Hanle Shang, Xianghui Zhao, Mingren Qu, Tao Peng, Beibei Guo, Yiqing Hu, Xiaozhen Song

**Affiliations:** Jiangxi Province Key Laboratory of Animal Nutrition/Engineering Research Center of Feed Development, College of Animal Science and Technology, Jiangxi Agricultural University, Nanchang, China

**Keywords:** *Puerarin*, beef cattle, heat stress, meat quality, muscle fiber type

## Abstract

The experiment was conducted to investigate the effects of dietary supplementation with *Puerarin* on meat quality, muscle antioxidant ability, and muscle fiber characteristics of beef cattle under a hot environment in summer. Thirty-two 15 ± 1.5-month-old Jinjiang bulls (291.65 ± 8.84 kg) were randomly divided into four groups with dietary *Puerarin* at 0 (control), 200 (Pue200), 400 (Pue400), and 800 (Pue800) mg/kg in the feed concentrate (*n* = 8). The feeding trial lasted for 60 days after a 10-day adaptation period (July 1–September 8); the average values of temperature, relative humidity and temperature, and humidity index were 30.68°C, 68.05%, and 81.81, respectively. The growth performance on day 40 of the experiment period was calculated. After 60 days' experimental period, four Jinjiang cattle per treatment from the control group, Pue400 group, and Pue800 group were slaughtered. Compared with the control group, the Pue400 and Pue800 groups improved the growth performance of beef cattle; the Pue800 group elevated the activities of superoxide dismutase, total antioxidant capacity, and glutathione peroxidase in the *M. longissimus thoracis* (LT) muscle. In the control group, the cell membrane was incomplete, and most of the mitochondria were elongated and in a fission state, while in the Pue400 and Pue800 groups, the cell membrane was clear and complete, and the mitochondria presented with round and oval shapes. Compared with the control group, the Pue400 and Pue800 groups reduced the shear force of the LT muscle, and the Pue400 group decreased the muscle fiber diameter and the myosin heavy-chain (MyHC)-IIb gene expression. Furthermore, the Pue400 and Pue800 groups decreased the ratio of AMP/ATP, the Pue800 group reduced the AMP-activated protein kinase α2 mRNA expression, and the Pue400 group improved the nuclear respiratory factor 1 mRNA expression. These results indicated that dietary supplementation with *Puerarin* might be beneficial to the meat quality of heat-stressed beef cattle by improving muscle antioxidant ability and reducing the MyHC-IIb muscle fiber composition. Based on the results of this study, we recommended 400 mg/kg *Puerarin* in the feed concentrate of beef cattle (~300 kg) for mitigation of heat stress.

## Introduction

Heat stress caused by high temperature is one of the most critical environmental stressors challenging cattle production (1), particularly during the hot-humid season in subtropical regions such as South China. The oxidative stress induced by heat stress can impair cell membrane and mitochondrial integrity through lipid peroxidation, which adversely affects the muscle protein functionality and the sensory, nutritional, and shelf-life quality of animal products (2, 3). Moreover, muscle fiber is an important part of the muscle; the quantity and type of muscle fiber are the keys to determine meat quality. Generally, muscle fiber is classified into four categories: myosin heavy-chain (MyHC)-I (slow-oxidative), MyHC-IIa (fast-oxidative), MyHC-IIx (intermediary to MyHC-IIa and IIb), and MyHC-IIb (fast-glycolytic) (4). They can influence meat quality due to their different activities of glycolytic enzymes and contents of glycogen and lipid (5). Notably, previous studies showed that acute or chronic heat stress resulted in decreased slow muscle fiber composition and ultimately led to poor meat quality (6, 7). Therefore, heat stress mitigation is a significant demand for beef cattle welfare and an important guarantee for high-quality beef production.

Unraveling the heat stress-responsive mechanisms in beef cattle is of great importance to the development of active agents against heat stress. AMP-activated protein kinase (AMPK) is a serine/threonine kinase that plays a crucial role in regulating cellular and whole-body energy homeostasis under physiological stress conditions (8). When the body suffers from heat stress, ATP consumption increases, subsequently resulting in elevation of AMP/ATP ratio and further activating AMPK *via* phosphorylation of αThr^172^ (9, 10). AMPK can also be activated by cellular reactive oxygen species (ROS) and intracellular calcium (11). Once activated, AMPK phosphorylates a range of substrates to preserve energy expenditure by switching from anabolic to catabolic pathway (8). Among all the AMPK-mediated regulation pathways of energy homeostasis maintenance in response to stress, what meat scientists valued most is the regulation of skeletal muscle energy metabolism (3). Moreover, the activation of AMPK has been proven to cause muscle to assume a faster-contracting and more glycolytic property (12). Therefore, regulating the activity of AMPK may effectively relieve the negative impact of heat stress on beef quality.

*Puerarin* is a natural active isoflavone extracted from the traditional Chinese medicine (TCM) *Radix Puerarin* and has a wide range of functions, including anti-oxidative, anti-inflammation, and anti-apoptosis (13, 14). It has been reported that *Puerarin* could attenuate heat stress by suppressing ROS production and upregulating heat shock protein-72 expression in bovine Sertoli cells (15). Besides, *Puerarin* could alleviate autophagy by inhibiting the AMPK–mammalian target of rapamycin (mTOR)–Unc 51 like autophagy activating kinase 1 (ULK1) signaling pathway (16). A previous study showed that *Puerarin* was used as a superior animal feed additive, natural health food, and effective medicine in Asian countries (17). However, little attention has been paid to *Puerarin*'s improvement effects on the meat quality of heat-stressed beef cattle, and whether this improvement is associated with the changes in muscle antioxidant ability and muscle fiber characteristics is still unclear. Therefore, this experiment was intended to study the effects of *Puerarin* on the meat quality, muscle antioxidant ability, muscle fiber characteristics, and AMPK pathway of beef cattle under high temperature in summer.

## Materials and Methods

This experiment was approved by the Committee for the Care and Use of Experimental Animals at Jiangxi Agricultural University (JXAULL-20190015).

### Preparation of *Puerarin*

*Puerarin* preparation (purity N 98.1%) was provided by the Department of Pharmaceutical Chemistry, Guangxi Medical University (Nanning, China). *Puerarin* is a white powder extracted from *R. Puerarin*. In these experimental diets, *Puerarin* was first mixed with the premix in a certain proportion and then formulated into a concentrate.

### Animal Treatments and Experimental Diets

Thirty-two 15 ± 1.5-month-old healthy and uncastrated Jinjiang bulls (291.65 ± 8.84 kg) were randomly divided into four groups. Jinjiang cattle are a breed of Chinese indigenous beef cattle that are bred in the northwest of the Jiangxi Province. Dietary treatments were basal diet without any additive (control), basal diet + 200 mg/kg *Puerarin* in the feed concentrate (Pue200), basal diet + 400 mg/kg *Puerarin* in the feed concentrate (Pue400), and basal diet + 800 mg/kg *Puerarin* in the feed concentrate (Pue800). Each treatment consisted of eight replicates (pen) with one beef cattle per pen. Animals were housed indoors with solid concrete floor pens (1.25 m × 2 m). The feeding trial lasted for 60 days after a 10-day adaptation period (July 1–September 8; the average values of temperature, relative humidity and temperature, and humidity index were 30.68°C, 68.05%, and 81.81, respectively). According to the Chinese Feeding Standard for beef cattle (NY/T 815-2004), the basal diet was designed and met the nutrient requirements of 0.5 kg daily gain of 300 kg male cattle. The ingredient composition and nutrient levels of the basal diet were shown in Table 1. In this study, the level of *Puerarin* supplemented in the basal diet was referred to the study of Zhao et al. (18), who reported that the crossbred steers (Chinese Yellow × Augus, 565.2 ± 31.2 kg) fed a 90% concentrate diet supplemented with daidzein (500 mg/kg concentrate). *Puerarin* is a structural analog of daidzein. Based on these data, it can be calculated that the equal amount of *Puerarin* is 908 mg/kg basal diet in 565 kg of beef cattle. Therefore, we hypothesized that 291 kg of Jinjiang cattle could be fed 468 mg/kg *Puerarin* in basal diet. No antibiotic was included in the diets. The diet was provided twice daily (06:00, 15:30). All animals were offered feed and water *ad libitum*.

**Table 1:** Composition and nutrient levels of the basal diet (air-dry basis, %).

**Ingredients**	**Content**	**Nutrient levels**	**Content**
Rice straw	20	DM	89.42
Brewer's grains	20	CP	11.19
Wheat	56.5	Ash	7.80
NaCl	0.5	NDF	30.08
NaHCO_3_	1.0	ADF	15.18
Premix*^a^*	2.0	NE_mf_/(MJ/kg)*^b^*	5.45
Amount	100.0	P	0.67
		Ca	1.11

a *The premix provided per kilogram of diet: 3,200 mg of iron as iron sulfate, 1,500 mg of manganese as manganous oxide, 2,000 mg of zinc as zinc oxide, 650 mg of copper as copper sulfate, 35 mg of iodate as calcium iodate, 10 mg of selenium as sodium selenite, 10 mg of cobalt as cobalt chloride, 130 g of calcium as calcium carbonate, 30 g of phosphorus as calcium hydrogen phosphate, 45 mg retinyl acetate, 40 μg cholecalciferol, and 3.0 mg DL-α-tocopheryl acetate*.

b *NE_mf_ were calculated values, while others were measured values. DM, dry matter; CP, crude protein; NDF, neutral detergent fiber; ADF, acid detergent fiber; NEmf, combined net energy; P, phosphorus; Ca, calcium*.

### Growth Performance

Due to this study being conducted to investigate the effects of heat stress on meat quality of beef cattle, we recorded final body weight on day 40 of the experiment period to avoid the pre-slaughter stress induced by weighing. Therefore, the initial body weight (on the first day of the experiment period), final body weight (on day 40 of the experiment period), and dry matter intake of beef cattle in each replicate were recorded to calculate average daily gain (ADG), average dry matter intake (ADMI), and the ratio of feed to gain (F/G). ADG = (final body weights per replicate – initial body weights per replicate)/40 days, ADMI = dry matter intake per replicate/40 days, F/G = ADMI/ADG.

### Sample Collection

At the end of the experiment, based on the earlier period data (day 1–40 of the experiment period) of growth performance, we found that the Pue200 group reduced the ADG and ADMI of beef cattle numerically compared with the control group, while the Pue400 and Pue800 groups significantly improved the ADG and ADMI of beef cattle. Therefore, four beef cattle per treatment with one cattle per pen with medium body weight were selected from the control, Pue400, and Pue800 groups for further analyses. A total of 12 beef cattle were gently led to the slaughterhouse on foot to avoid stress (the distance between feedlot and slaughterhouse was 0.7 km). At the day of slaughter, the average temperature and humidity index (THI) was 78.3.

All animals were sacrificed *via* electrical stunning, followed by jugular vein exsanguination. Immediately, the *Musculus longissimus thoracis* (LT) muscle sampled at the last rib of the left carcass was placed in liquid nitrogen to analyze the antioxidant index, adenosine phosphates, and the expression of genes mRNA. Then, the LT muscle samples were cut into shaped strips (1 cm × 1 cm × 1 cm), parallel to the muscle fiber direction, and placed in a 4% formaldehyde solution for the analysis of muscle fiber morphology. The other LT muscle samples were cut into shaped strips (1 mm × 1 mm × 1 mm), parallel to the muscle fiber direction, placed in a pre-cooled 2.5% glutaraldehyde for 2 h, and stored in the refrigerator at 4°C until for the analysis of skeletal muscle ultrastructure. At 30-min postmortem, the LT muscles from the last rib of the left carcass were removed, trimmed of subcutaneous fat and connective tissue, and vacuum-packed at 4°C for the measurement of meat quality.

### Antioxidant Index Analyses

For biochemical assays, 0.5 g frozen LT muscle samples were ice bath homogenized in 4.5 ml ice-cold physiological saline for 1 min and then centrifuged (2,700 g, 4°C, 10 min). The supernatants were then subjected to the measurements of the content of malondialdehyde (MDA), the activities of superoxide dismutase (SOD) and glutathione peroxidase (GSH-PX), the total antioxidant capacity (T-AOC). The concentration of total protein in the LT muscle was determined by TP kit (Nanjing Jiancheng Bioengineering Institute, Nangjing, China) according to the instructions of the manufacturer. All of those antioxidant indices in the LT muscle were determined by commercial MDA, SOD, GSH-PX, and T-AOC kits (Nanjing Jiancheng Bioengineering Institute) and were normalized by total protein concentration in the LT muscle, respectively, according to the instructions of the manufacturer. The ROS content was determined by ROS kit (Nanjing Jiancheng Bioengineering Institute, Nangjing, China) according to the instructions of the manufacturer. The 2′,7′-dichlorofluorescin diacetate (DCFH-DA) is the most commonly used sensitive ROS probe.

### Meat Quality

Muscle pH_45min_ and pH_24h_ were measured by using a pH electrode (HI99163N, Hanna, Padova, Italy). Each chop was measured three times at different areas, and the average value was obtained.

Meat color was measured using a spectrocolorimeter (WSC-S, Shanghai, China) at 24 h postmortem. Mean CIE L^*^ (lightness), a^*^ (redness), and b^*^ (yellowness) values were collected from three different locations of the chops using freshly cut surface after being exposed to air for 20 min.

For the determination of cooking loss, at 48 h postmortem, samples of LT muscle (6 cm × 6 cm × 4 cm) were vacuum-packed in individual polyethylene vacuum bags and cooked in a water bath at 80°C to reach an internal temperature of 70°C. Then, the samples within their packaging bags were cooled in running water to room temperature, wiped with absorbent paper to remove residual moisture, and reweighed to calculate cooking loss. Then, the cooked samples were cut into shaped strips (1 cm × 1 cm × 3 cm), parallel to the muscle fiber direction. Warner–Bratzler shear force was measured using a C-LM3B shear apparatus (Northeast Agricultural University, Harbin, China) with a load cell of 15 kg and a crosshead speed of 200 mm/min. Six replicates of each sample were measured.

### Muscle Fiber Morphology

The LT muscle samples in 4% formaldehyde solution were taken out and then dehydrated, paraffin-embedded, sliced, hematoxylin–eosin-stained, and sealed. The sections were taken photographs with a microscope (Motic BA210; Motic Medical Diagnostic Systems, Co., Ltd., Xiamen, China) at a magnification of 400×. Motic Images Advanced 3.2 software was used to analyze pictures, including muscle fiber diameter and density. Five views were captured in each section.

### Transmission Electron Microscopy

The LT muscle samples in 2.5% glutaraldehyde were taken out and then washed with 0.1 mol/L phosphate buffer followed by postfixation of 1% osmium tetraoxide. Samples were then washed with 0.1 mol/L phosphate buffer again, dehydrated by gradient of increasing concentrations of alcohol, embedded with epon resin, and sliced. Transmission electron microscopy (TEM) images were obtained on a Hitachi H-7100 transmission electron microscope.

### Adenosine Phosphate Analyses

The levels of adenosine phosphates (ATP, ADP, and AMP) in the LT muscle were determined by high-performance liquid chromatography (HPLC) according to the method of Li et al. (19). Briefly, 0.5 g of frozen muscle sample was homogenized in 2.5 ml of 7% ice-cold perchloric acid at 13,500 rpm for 30 s in an ice bath and then centrifuged (15,000 × g, 4°C, 10 min). The supernatant (850 μl) was then neutralized with 0.85 M KOH (850 μl) and centrifuged again (15,000 × g, 4°C, 10 min) to remove KClO_4_. The neutralized supernatant was filtered through a 0.45-μm filter before injection into a Waters-2695 Alliance HPLC system (Waters, Milford, MA, USA). The column was a Kromasil 5 μm C_18_, 250 × 4.6 mm (Feinano, Tianjin, China). The chromatographic conditions were as follows: mobile phase A, HPLC grade methanol; mobile phase B, phosphate buffer (2.5 mM tetra-butylammonium hydrogen sulfate, 0.04 M potassium dihydrogen orthophosphate, 0.06 M dipotassium hydrogen orthophosphate, pH 7.0), filtered through a 0.45-μm membrane; mobile A/mobile B, 13.5%/86.5%. The column temperature was set at 30°C, the injection volume was 10 μl, and UV detection was at 254 nm. The characteristic running time was 15 min, the flow rate was maintained at 1.0 ml/min, and sample measurement was set to auto-sequence injection. Peaks were identified and quantified using standard curves.

### Total RNA Isolation and mRNA Expression Analyses

Total RNA was isolated from the LT sample using the phenol and guanidine isothiocyanate-based TRIzol reagent (Invitrogen, USA) according to the manufacturer's protocol. The purity and quantity of total RNA were measured by a NanoDrop 1000 spectrophotometer (Thermo Scientific, Wilmington, DE, USA) at 260 and 280 nm. Total RNA was treated with DNase I (Takara Biotechnology Co. Ltd., Dalian, China) to remove DNA and transcribed to cDNA using a PrimeScript RT Master Mix kit (Takara Biotechnology Co. Ltd., China) following the manufacturer's instructions. The mRNA expressions were determined and calculated according to the method of Li et al. (20). Briefly, real-time PCR was carried out in optical 96-well plates on an ABI 7500 Real-Time PCR System (Applied Biosystems, Foster City, CA, USA) and SYBR Premix Ex Taq Kit (Takara Biotechnology Co., Ltd., China). Primers used for real-time PCR are presented in Table 2 and were synthesized by Invitrogen. The amplification was performed in a total volume of 20 μl, containing 10 μl of SYBR Premix Ex Taq, 0.4 μl of each primer (10 μM), 6.8 μl of sterilized double-distilled water, and 2 μl of cDNA. The program was as follows: 95°C for 30 s, followed by 40 cycles of 95°C for 5 s, 58°C for 31 s, and 70°C for 30 s, and collected the fluorescence signal at 58°C. The amplification of the glyceraldehyde-3-phosphate dehydrogenase (GAPDH) housekeeping genes was used for each sample to normalize the expression of the selected genes. Relative gene expression was calculated using the 2^−ΔΔCt^ method.

**Table 2:** The primer sequences of genes.

**Gene**	**Primer sequence (5^**′**^-3^**′**^)**	**Number**	**Product size (bp)**
LKB1	F: CACCGAGGTCATCTACCAGC	XM_024995125.1	114
	R: GAGTCCAGCACCTCCTTCAC		
AMPKα2	F: TGAGAAGCAGAAGCACGACG	NM_001205605.1	113
	R: GGCCTGTCAATTGATGCTCT		
PGC-1α	F: TGCAGTACACATCAGCCTCA	NM_177945.3	95
	R: TGCCAGGAGTTTGGTTGTGAT		
Nrf1	F: AAACTGGGCCACGTTACAGG	NM_001098002.2	175
	R: TTTTATTGCCCACCCCTGCC		
MyHC-I	F: CAAGGAGCTTCAGGCACGTA	NM_174727.1	179
	R: CGCGCTTCTTGTTCATCTCG		
MyHC-IIa	F: AGACTCTCAAGAGGGACGCT	XM_010816053.3	77
	R: CCTGGAAGTGAGACGGTTCC		
MyHC-IIx	F: CTGAGGAACGGGCTGACATT	NM_174117.1	164
	R: AGTACAAAACAGAGTGACAAAGATT		
MyHC-IIb	F: GTCGGGCTGTACCAGAAGTC	XM_002695806.5	74
	R: CCCTCTTCAGCACTTGGACC		
GAPDH	F: GAAGGTCGGAGTGAACGGAT	NM_001034034.2	180
	R: TTCTCTGCCTTGACTGTGCC		

*LKB1, receptor serine/threonine kinase 1; AMPKα2, AMP-activated protein kinase α2; PGC-1α, peroxisome proliferator-activated receptor γ coactivator 1α; Nrf1, nuclear respiratory factor 1; MyHC, myosin heavy chain; GAPDH, glyceraldehyde-3-phosphate dehydrogenase*.

### Statistical Analyses

All data analyses were statistically analyzed by one-way ANOVA with SPSS statistical software (Ver. 20 for windows, SPSS). All data were normally distributed *via* the Shapiro–Wilk test. Tukey multiple range test was used to compare differences among the treatment groups. The level of statistical significance was present at *P* < 0.05. Values were expressed as mean ± SE.

## Results

### Growth Performance

As shown in Table 3, compared with the control and Pue200 group, dietary supplementation with 400 and 800 mg/kg *Puerarin* improved the ADG of beef cattle (*P* < 0.05). The ADMI of beef cattle in the Pue800 group was higher than that in the control group (*P* < 0.05). However, there was no difference in the initial and final body weights and F/G of cattle among all treatments.

**Table 3:** Effects of *Puerarin* on growth performance of beef cattle under hot environment.

	**Groups**				
**Item**	**Control**	**Pue200**	**Pue400**	**Pue800**	***P-*value**
Initial body weight (kg)	298.86 ± 14.34	294.50 ± 21.46	280.40 ± 25.36	290.86 ± 16.54	0.915
Final body weight (kg)	318.43 ± 14.32	310.80 ± 16.45	308.60 ± 23.16	318.29 ± 7.90	0.965
ADG (kg/day)	0.49 ± 0.02^b^	0.41 ± 0.03^b^	0.71 ± 0.07^a^	0.69 ± 0.07^a^	0.002
ADMI (kg/day)	4.53 ± 0.25^bc^	4.02 ± 0.24^c^	5.41 ± 0.39^ab^	5.70 ± 0.30^a^	0.003
F/G (kg/kg)	9.34 ± 0.63	10.05 ± 0.68	7.85 ± 0.58	8.59 ± 0.51	0.125

a, b *Means within a row with no common superscript differ significantly (P < 0.05)*.

*All traits in this table were analyzed with cattle as the experimental unit (n = 8)*.

*ADG, average daily gain; ADMI, average dry matter intake; F/G, feed-to-gain ratio*.

### Antioxidant Index

As presented in Table 4, the Pue800 group tended to reduce the level of ROS compared with the control group (*P* = 0.051). The SOD activity in the Pue400 and Pue800 groups was higher than that in the control group (*P* < 0.05). In comparison to the control group, the Pue800 group increased the activities of T-AOC and GSH-PX in the LT muscle (*P* < 0.05). However, an insignificant effect was presented on the MDA content.

**Table 4:** Effects of *Puerarin* on antioxidant index in *M. longissimus thoracis* of beef cattle under hot environment.

	**Groups**			
**Item**	**Control**	**Pue400**	**Pue800**	***P-*value**
ROS (fluorescence value/mg prot)	52.72 ± 1.51	51.60 ± 3.17	43.07 ± 2.72	0.051
SOD (U/mg prot)	6.88 ± 0.31^b^	8.36 ± 0.27^a^	8.34 ± 0.40^a^	0.017
T-AOC (U/mg prot)	0.87 ± 0.04^b^	0.99 ± 0.08^ab^	1.13 ± 0.06^a^	0.042
GSH-PX (U/mg prot)	55.98 ± 1.69^b^	56.65 ± 2.55^b^	66.23 ± 2.42^a^	0.018
MDA (nmol/mg prot)	0.48 ± 0.02	0.42 ± 0.02	0.43 ± 0.03	0.176

a, b *Means within a row with no common superscript differ significantly (P < 0.05)*.

*All traits in this table were analyzed with cattle as the experimental unit (n = 4)*.

*ROS, reactive oxygen species; SOD, superoxide dismutase; T-AOC, total antioxidant capacity; GSH-PX, glutathione peroxidase; MDA, malondialdehyde*.

### Transmission Electron Microscopy

**Figure 1:**
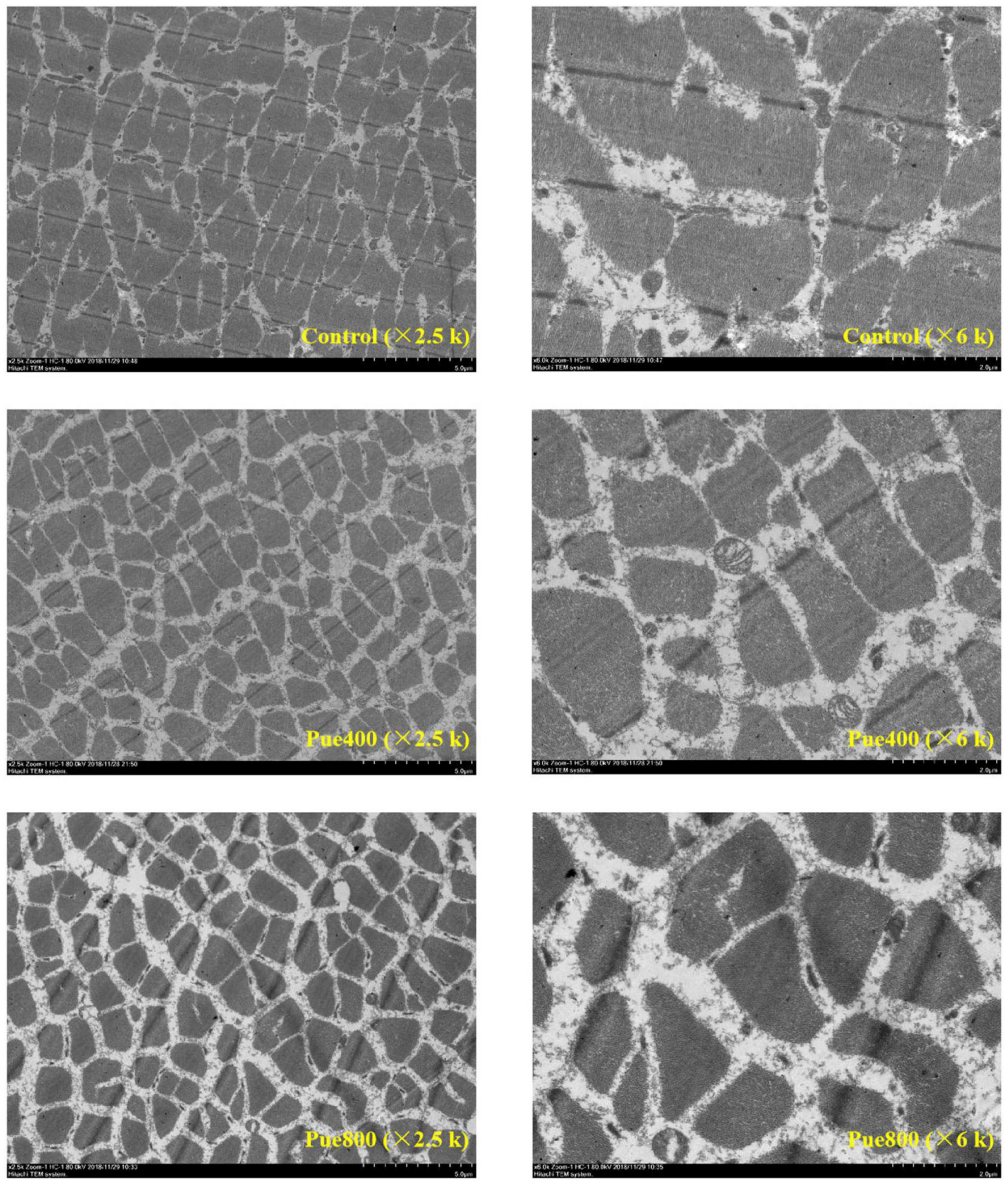
Effects of *Puerarin* on the skeletal muscle ultrastructure in *M. longissimus thoracis* of beef cattle under hot environment.

As shown in Figure 1, in the control group, the skeletal muscle cell boundaries were not clearly visible and had an incomplete membrane. Besides, the mitochondrial structure was deformed, most of the mitochondria were elongated and in a fission state. In the Pue400 and Pue800 groups, the cell membrane was clear and complete, and the mitochondrial structure was standard, which presented with round and oval shapes. The mitochondrial cristae in the Pue400 group were normal with clear structures, whereas this was not the case in the Pue800 group.

### Meat Quality

As shown in Table 5, diet supplemented with 400 and 800 mg/kg *Puerarin* reduced the shear force of LT muscle compared with the control group (*P* < 0.05), while there was no significant difference in pH_45min_ value, pH_24h_ value, lightness (L^*^) values, redness (a^*^) values, yellowness (b^*^) values, and cooking loss of the LT muscle among groups.

**Table 5:** Effects of *Puerarin* on the meat quality in *M. longissimus thoracis* of beef cattle under hot environment.

	**Groups**			
**Item**	**Control**	**Pue400**	**Pue800**	***P-*value**
pH_45min_	6.59 ± 0.07	6.60 ± 0.21	6.61 ± 0.10	0.991
pH_24h_	5.42 ± 0.05	5.68 ± 0.01	5.55 ± 0.15	0.269
Lightness, L^*^	0.46 ± 0.04	0.51 ± 0.18	0.43 ± 0.03	0.606
Redness, a^*^	3.07 ± 0.45	2.91 ± 0.38	3.09 ± 0.38	0.793
Yellowness, b^*^	1.07 ± 0.02	1.19 ± 0.18	0.83 ± 0.09	0.745
Cooking loss (%)	21.78 ± 4.79	18.14 ± 5.09	17.07 ± 3.21	0.335
Shear force (N)	80.97 ± 0.77^b^	58.83 ± 5.84^a^	75.28 ± 1.48^a^	0.014

a, b *Means within a row with no common superscript differ significantly (P < 0.05)*.

*All traits in this table were analyzed with cattle as the experimental unit (n = 4)*.

### Muscle Fiber Morphology

**Figure 2:**
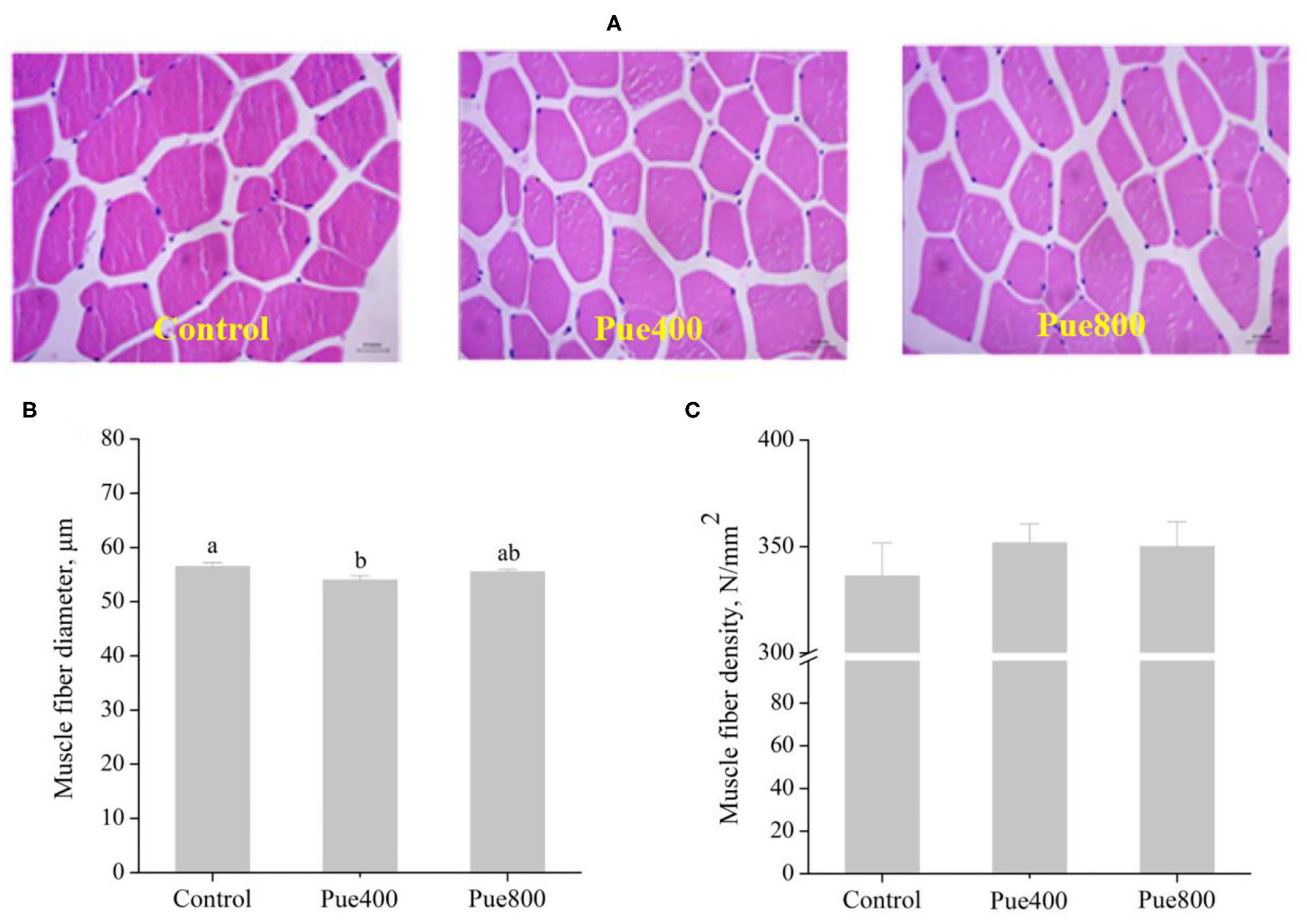
Effects of *Puerarin* on muscle fiber morphology **(A)**, muscle fiber diameter **(B)**, and muscle fiber density **(C)** in *M. longissimus thoracis* of beef cattle under hot environment.

As shown in Figure 2, diet supplemented with 400 mg/kg *Puerarin* reduced the diameter (Figure 2B) of the LT muscle compared with the control group (*P* < 0.05). However, there was no difference in the muscle fiber density (Figure 2C) among groups.

### Muscle Fiber Type

**Figure 3:**
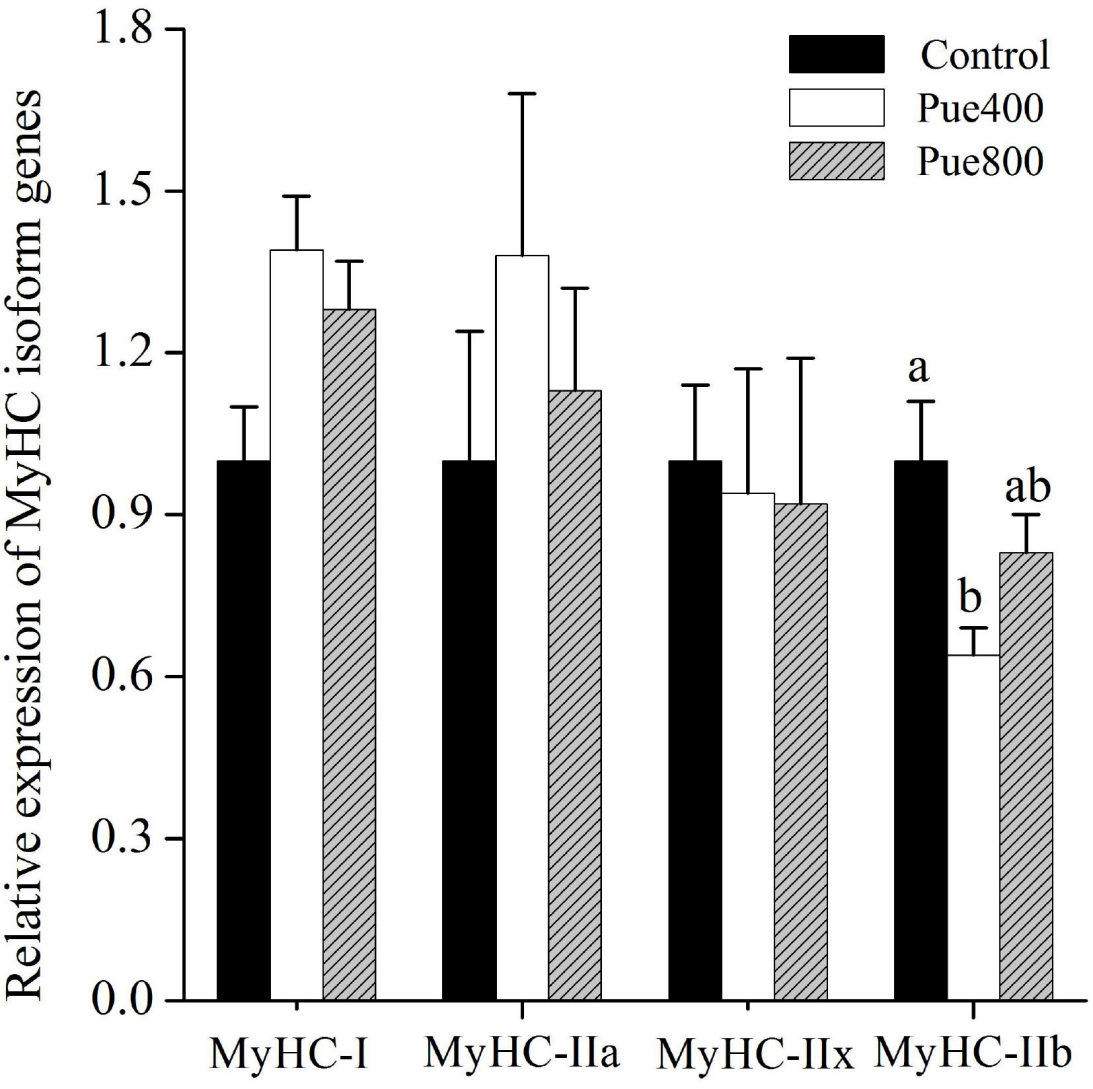
Effects of *Puerarin* on the relative mRNA expressions of myosin heavy-chain (MyHC) isoform genes in *M. longissimus thoracis* of beef cattle under hot environment (*n* = 4). (■): dietary supplementation with 0 mg/kg *Puerarin* in the feed concentrate; (□): dietary supplementation with 400 mg/kg *Puerarin* in the feed concentrate; (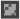): dietary supplementation with 800 mg/kg *Puerarin* in the feed concentrate.

As shown in Figure 3, diet supplemented with 400 mg/kg *Puerarin* reduced the MyHC-IIb gene expression compared with the control group (*P* < 0.05). No difference was noticed about the mRNA expression of MyHC-I, MyHC-IIa, and MyHC-IIx among treatments.

### Adenosine Nucleotides

As presented in Table 6, diet supplemented with 400 mg/kg *Puerarin* improved the content of ATP compared with the control group (*P* < 0.05), and the ratio of AMP/ATP in the Pue400 and Pue800 groups were lower than those in the control group (*P* < 0.05). However, no difference was noticed about the contents of ADP and AMP among groups.

**Table 6:** Effects of *Puerarin* on the contents of adenosine nucleotides in *M. longissimus thoracis* of beef cattle under hot environment.

	**Groups**			
**Item**	**Control**	**Pue400**	**Pue800**	***P-*value**
ATP (mg/g)	1.08 ± 0.06^a^	1.91 ± 0.04^b^	1.20 ± 0.22^a^	0.010
ADP (mg/g)	0.76 ± 0.04	0.84 ± 0.14	0.98 ± 0.04	0.288
AMP (mg/g)	2.13 ± 0.22	1.49 ± 0.02	1.87 ± 0.32	0.082
AMP/ATP	2.10 ± 0.13^a^	0.78 ± 0.03^b^	1.41 ± 0.29^b^	0.007

a, b *Means within a row with no common superscript differ significantly (P < 0.05)*.

*All traits in this table were analyzed with cattle as the experimental unit (n = 4)*.

### AMPK Signal Pathway-Related Gene Expressions

**Figure 4:**
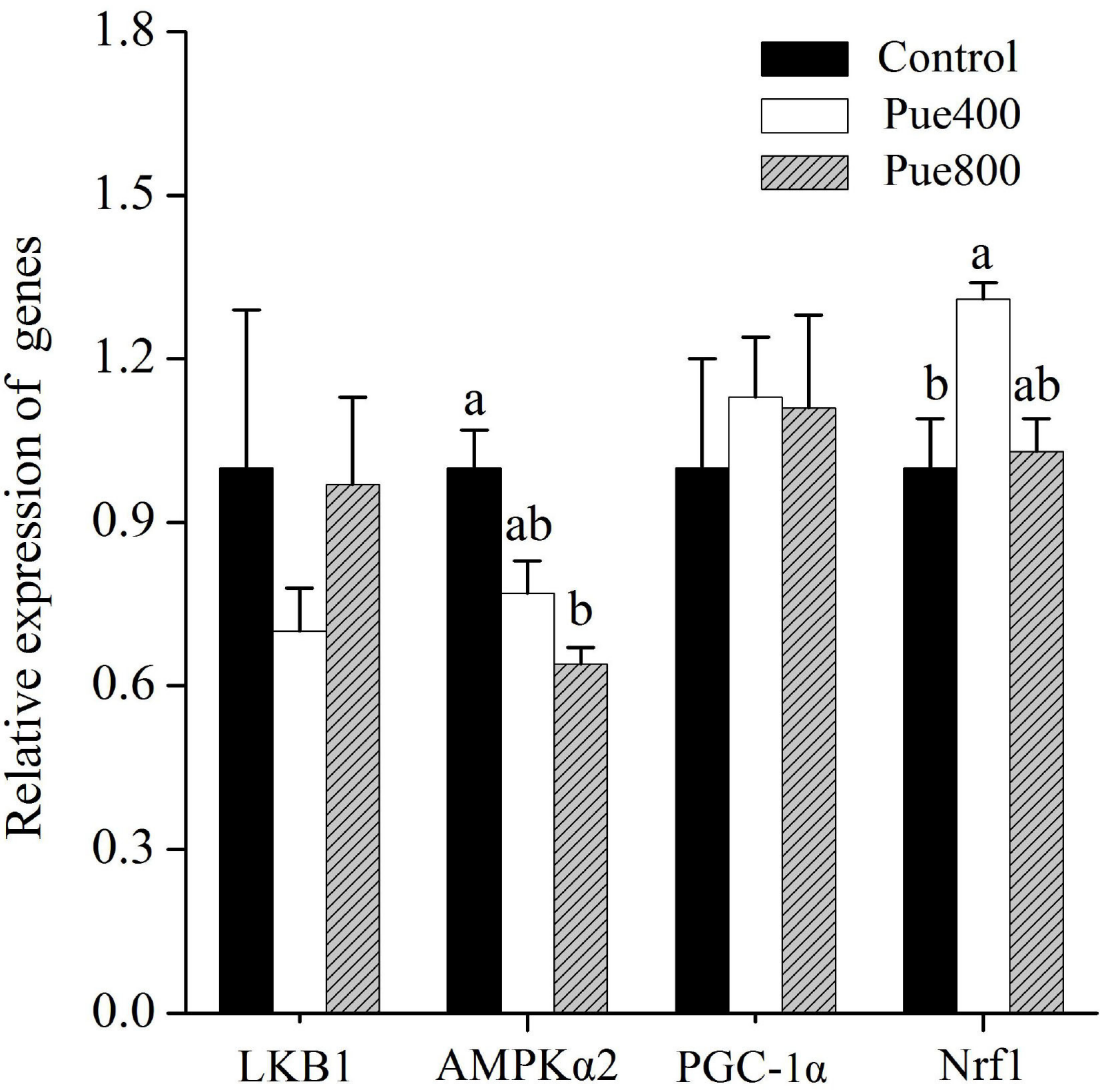
Effects of *Puerarin* on the relative mRNA expression of LKB1, AMPKα2, PGC-1α, and Nrf1 in *M. longissimus thoracis* of beef cattle under hot environment (*n* = 4). (■): dietary supplementation with 0 mg/kg *Puerarin* in the feed concentrate; (□): dietary supplementation with 400 mg/kg *Puerarin* in the feed concentrate; (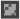): dietary supplementation with 800 mg/kg *Puerarin* in the feed concentrate. LKB1, receptor serine/threonine kinase 1; AMPKα2, adenosine monophosphate-activated protein kinase α2; PGC-1α, peroxisome proliferator-activated receptor-γ coactivator-1α; Nrf1, nuclear respiratory factor 1.

As shown in Figure 4, compared with the control group, the Pue800 group reduced the mRNA expression of AMPKα2 (*P* < 0.05). Moreover, the Pue400 group improved the mRNA expression of nuclear respiratory factor 1 (Nrf1) (*P* < 0.05). However, an insignificant effect was presented on the receptor serine/threonine kinase 1 [liver kinase B1 (LKB1)] and peroxisome proliferator-activated receptor γ coactivator 1α (PGC-1α) gene expression.

## Discussion

The results of our previous study showed that the average daily THI values during the experimental period were higher than 79 for 54 out of 60 days (unpublished data), which indicated that the experimental beef cattle were in a state of heat stress according to the report of Livestock Conservation, Inc. (LCI) (21). It is widely known that heat stress leads to the decline of production performance, which has been suggested to be partly caused by the reduction of dry matter intake (22). In this study, diet supplemented with *Puerarin* improved the ADG of beef cattle, which might be partly attributed to the increased ADMI induced by *Puerarin* supplementation.

Numerous studies show that farm animals exposed to a hot environment are also related to an occurrence of oxidative stress and further result in a disorder of free radical metabolism and excessive production of ROS (2, 23). Subsequently, the high level of muscle ROS may increase the risk of oxidative reactions during the processes involved in the conversion of muscle into meat. One previous study found that pretreatment with *Puerarin* protected bovine Sertoli cells (bSCs) from heat stress by suppressing ROS production and changing the SOD, catalase (CAT), and GSH-PX activities and MDA content (15). Another study reported that *Puerarin* supplementation reduced the level of ROS and increased the activities of SOD and CAT in the kidney of diabetic mice (24). Similarly, in this study, dietary supplementation with *Puerarin* markedly increased the activities of SOD, GSH-PX, and T-AOC and reduced the content of MDA in the LT muscle of heat-stressed beef cattle. There was little research conducted on the effect of *Puerarin* on the antioxidant capacity of beef cattle. For ruminant, the isoflavones are readily hydrolyzed by rumen microbes, and the major metabolic transformation of isoflavones is performed in the rumen (25). There is evidence that *Puerarin* can be transformed into daidzein by bacterial enzymes in the large intestine of rats (26), and daidzein can be partly metabolized to equol by ruminal microorganisms (25). Daidzein and equol also have intrinsic antioxidant activities (27). Therefore, we speculated that *Puerarin* might have antioxidant effects for beef cattle in the form of daidzein, equol, and itself.

The tenderness of meat is the most important quality attribute influencing the consumer decision to purchase. There are complex interactions among various biochemical traits across multiple muscles affecting meat tenderness. One of the key events is the mitochondrial function. In skeletal muscle, mitochondria are abundant, and the power houses of cells function in muscle energy metabolism. Meanwhile, mitochondria are the main sites for ROS production; accumulation of damaged or dysfunctional mitochondria promotes an increase in ROS production (28). Mitochondrial functions are intrinsically linked to their morphology and membrane ultrastructure (29). Under physiological conditions, mitochondria are dynamic organelles that undergo permanent fission and fusion. While under heat stress conditions, the hypothalamic–pituitary–adrenal (HPA) axis was activated, which further induces calcium overload and promotes mitochondrial fission through phosphorylation of dynamin-related protein 1 (DRP1) (30). Our results were consistent with the theory that showed that heat stress disturbs the integrity of skeletal muscle cell membrane and keeps the most mitochondria in a fission state, while dietary supplementation with 400 mg/kg *Puerarin* promoted the integrity of cell membrane and maintained the mitochondrial morphology in round and oval shapes. Consistent with our results, Chen et al. (31) reported that *Puerarin* could improve the function of mitochondria in the muscle of diabetic rats by upregulating mitochondrial biogenesis. These results suggested that *Puerarin* could attenuate oxidative stress induced by heat stress in beef cattle, which may help improve meat tenderness by inhibiting the oxidation of muscle proteins, enzymes, and lipid initiated by free radicals and protecting the structure and functional integrity of muscle mitochondria and enzymes (32). Consistently, our results showed that *Puerarin* supplementation decreased the shear force of the LT muscle compared with the control group.

Besides, long-term heat stress has been reported to increase the muscle fiber diameter and lead to a higher shear force of meat (33). Generally, the thinner and denser the muscle fiber, the more significant tenderness of the meat (34). Fortunately, in the current study, our results showed that dietary supplementation with *Puerarin* reduced the muscle fiber diameter of the LT muscle, which was also advantageous for increased tenderness. Many factors influence muscle fiber traits, among them, the muscle fiber type is one of the most pivotal factors with fine oxidative fibers and coarse glycolytic fiber (35). Previous studies reported that muscle with more slow-twitch type I fibers might improve tenderness in cattle (36), while an increasing proportion of MyHC-IIb fiber type in muscle usually lead to higher lightness and drip loss in raw meat (37). Correspondingly, in the present study, dietary supplementation with *Puerarin* decreased the mRNA expression of MyHC-IIb of the LT muscle in heat-stressed beef cattle. It has been accepted that heat stress stimulates the glycolysis process (38) and resulted in a decrease in slow muscle fiber composition (7). Therefore, we speculated that *Puerarin*'s modulation on muscle fiber type was dependent on heat stress relief. However, further investigation is required to determine the specific regulation mechanism of muscle fiber type transformation induced by *Puerarin*.

Previous research showed that AMPK played an essential role in promoting muscle to assume a faster-contracting, more glycolytic nature (12). Recent studies reported that *Puerarin* alleviated autophagy by inhibiting the phosphorylation of AMPK (16). Similarly, our results showed that dietary supplementation with *Puerarin* decreased the mRNA expression of AMPK. The AMP/ATP ratio, as an essential regulator of AMPK activity (9), was also reduced by dietary supplementation with *Puerarin*. In the summer, oxidative stress induced by heat exposure leads to mitochondrial damage and impairing ATP synthesis (39), which ultimately results in a higher AMP/ATP ratio. Fortunately, Xue et al. (40) reported that *Puerarin* could stabilize mitochondrial potential by protecting the mitochondrial function. Correspondingly, in this study, the decreased AMP/ATP ratio in the LT muscle might correlate with the increased content of ATP induced by the benefit of *Puerarin* on mitochondrial function, whereas LKB1, which is also an upstream activator of AMPK, did not vary significantly among treatments.

PGC-1α, as a transcriptional activator, is shown to upregulate the slow fiber gene expression. While Nrf1 is the downstream target gene of PGC-1α, it has also been reported to involve the transformation of muscle fiber type (41). In this study, dietary supplementation with *Puerarin* numerically increased the mRNA expression of PGC-1α, whereas it significantly increased the Nrf1 gene expression. Similarly, in C2C12 cells, treatment with *Puerarin* increased the protein expression levels of PGC-1α and Nrf1 (42). Therefore, we speculated that *Puerarin* affected muscle fiber type of beef cattle via regulating AMPK and Nrf1 gene expressions under heat stress.

## Conclusion

In conclusion, dietary supplementation with *Puerarin* could decrease the meat shear force of beef cattle under high temperature. Moreover, the improvement of meat quality was *via* improving muscle antioxidant ability and reducing the MyHC-IIb muscle fiber composition of the heat-stressed beef cattle. Based on the results of this study, we recommended 400 mg/kg *Puerarin* in the feed concentrate of beef cattle (~300 kg) for mitigation of heat stress.

## Data Availability Statement

The datasets presented in this article are not readily available because the study data are owned by Jiangxi Province Key Laboratory of Animal Nutrition. Access to these data would require additional approval beyond that of the authors. Requests to access these datasets should be directed to Dr. Yanjiao Li, yanjiaoli221@163.com.

## Ethics Statement

This experiment was approved by the Committee for the Care and Use of Experimental Animals at Jiangxi Agricultural University (JXAULL-20190015).

## Author Contributions

YL, HS, and XS designed the overall study. HS, XZ, MQ, TP, BG, and YH performed the experiments. YL and XS wrote the manuscript. All authors contributed to the article and approved the submitted version.

## Conflict of Interest

The authors declare that the research was conducted in the absence of any commercial or financial relationships that could be construed as a potential conflict of interest.
